# Psammomatoid ossifying fibroma in the frontal sinus: An intriguing clinical encounter—A detailed case report

**DOI:** 10.1002/cnr2.2063

**Published:** 2024-04-16

**Authors:** Pegah Babaheidarian, Parisa Mokhles, Saleh Mohebbi, Razieh Shahnazari, Nasser Karimi, Donya Ghazinia, Sina Karaji, Shahriar Shirzadi

**Affiliations:** ^1^ Department of Pathology School of Medicine, Iran University of Medical Sciences Tehran Iran; ^2^ Skull Base Research Center Five Sense Health Institute, School of Medicine, Iran University of Medical Sciences Tehran Iran; ^3^ Firoozabadi Clinical Research Development Unit (FACRDU), Department of Radiology, School of Medicine, Iran University of Medical Sciences Tehran Iran; ^4^ Eye and Skull Base Research Centers The Five Senses Institute, Rassoul Akram Hospital, Iran University of Medical Sciences Tehran Iran; ^5^ Department of Radiology School of Medicine, Iran University of Medical Sciences Tehran Iran; ^6^ Department of Radiology Hamedan University of Medical Sciences Hamedan Iran

**Keywords:** craniofacial lesion, frontal sinus tumor, juvenile psammomatoid, ossifying fibroma, surgical management

## Abstract

**Background:**

Psammomatoid ossifying fibroma (POF) is a rare craniofacial neoplasm, primarily affecting the maxillofacial region, and typically observed in adolescents and young adults. This case report presents a unique occurrence of POF in a 50‐year‐old male, defying the conventional age range and exhibiting an unusual anatomical location within the frontal sinus.

**Case:**

A 50‐year‐old male with a prior history of cecal adenocarcinoma and colectomy presented with left eye proptosis and new‐onset headaches. Imaging revealed a well‐defined calcified mass in the left frontal sinus, leading to a diagnosis of POF. Open surgical resection was performed to remove the tumor, and histopathological evaluation confirmed its diagnosis as psammomatoid ossifying fibroma. The patient exhibited no postoperative complications or signs of recurrence.

**Conclusion:**

This case underscores the diverse clinical presentations and diagnostic challenges associated with POF, emphasizing the importance of accurate diagnosis and multidisciplinary collaboration. Further research is needed to explore the genetic underpinnings and optimal management strategies for this intriguing condition.

## 1 INTRODUCTION

Ossifying fibromas, benign fibroosseous neoplasms that primarily impact the jaws and the craniofacial structure, manifest in three distinct clinicopathological variations. These include the odontogenic‐origin ossifying fibroma, known as cemento‐ossifying fibroma (COF), alongside two unique juvenile ossifying fibromas: juvenile trabecular ossifying fibroma (JTOF) and juvenile psammomatoid ossifying fibroma (JPOF). Psammomatoid ossifying fibroma (POF) is an infrequent tumor within the category of fibroosseous lesions of the craniofacial region. The rarity of JPOF makes it an uncommon finding in clinical practice. JPOF primarily affects individuals in their adolescent and young adult years. The reported mean patient age for JPOF typically falls within the range of 16–33 years, indicating a predilection for this age group. Notably, JPOF can manifest across a wide age spectrum, with reported cases occurring in patients as young as 3 months and as old as 72 years. This broad age range underscores the potential for JPOF to present in individuals beyond the typical juvenile age group.^1^

The systematic review encompassed an extensive period, ranging from the year 2000 to 2022, yielding valuable insights into juvenile psammomatoid ossifying fibroma (JPOF). The review elucidated that JPOF predominantly manifests within the confines of the maxillofacial region, with 44% of documented cases localized in the maxilla and 56% in the mandible. It is noteworthy that JPOF exhibits a proclivity for a younger demographic, with males being more prevalent among individuals below 14 years of age, while females tend to predominate among those older than 14 years. Radiographically, JPOF frequently presents as an expansive osteolytic lesion characterized by varying degrees of mineralization, often involving adjacent anatomical structures. Histopathologically, the hallmark feature of JPOF is its highly cellular stroma adorned with distinctive psammomatoid bodies. The therapeutic landscape for this condition is multifaceted, encompassing surgical excision or enucleation and curettage; however, it is pertinent to note that the specter of recurrence looms large, necessitating meticulous, long‐term surveillance. These findings underscore the imperative for a judicious and thorough approach to diagnosis and management of this unique craniofacial anomaly within the academic domain.^2^

In the case presented, we encounter a 50‐year‐old man who had a notable medical history, including a prior diagnosis of adenocarcinoma and colectomy performed approximately 17 years ago. His current complaint centered around left eye proptosis, prompting medical evaluation. This case brings to the forefront the intricacies of managing craniofacial lesions, underscoring the importance of accurate diagnosis and tailored interventions.

## 2 CASE PRESENTATION

In August 2023, a 50‐year‐old male patient presented to Rassoul Akram Hospital in Tehran, Iran, with a disfiguring proptosis in the left eye and new‐onset headaches. The patient reported a gradual development of proptosis over the past two decades. Notably, the patient's medical history included a cecal adenocarcinoma diagnosed in 2006, for which he underwent a colectomy. The patient reported no ocular pain or visual disturbances, and initial ophthalmic evaluation indicated a visual acuity of 10/10 in the right eye and 9/10 in the left eye, with no relative afferent pupillary defect observed.

**FIGURE 1:**
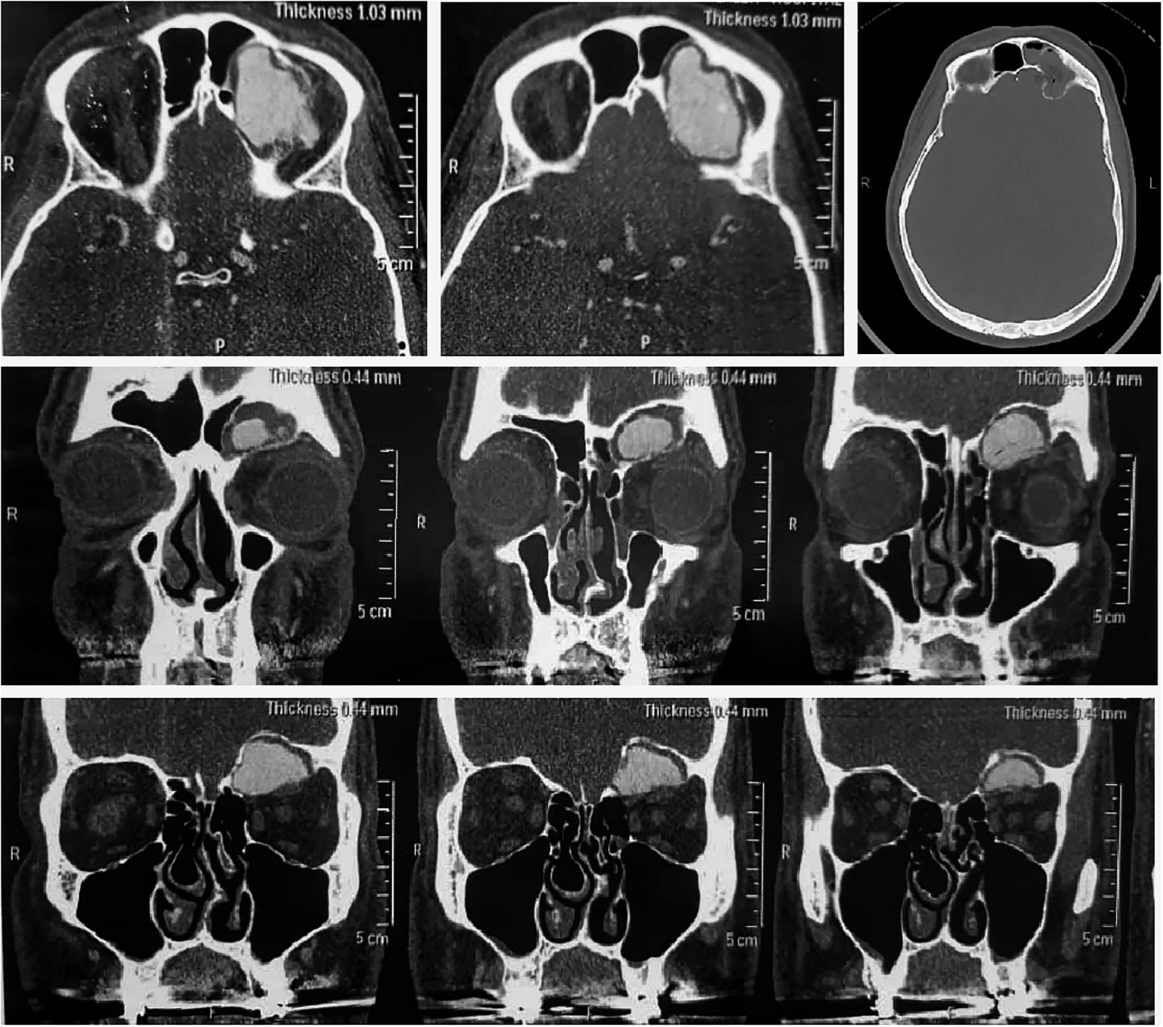
On the axial and coronal sequences of the CT scan with contrast, there is a well‐defined lytic expansile growth pattern with psammomatoid calcifications as small, round calcification in peripheral region of tumor in the left frontal sinus. There is no evidence of sclerotic margins or invasive features, cortical breakthrough. The images show significant contrast enhancement that is proposed the vascularity nature of the tumor.

Motility examinations revealed mild restriction in upward gaze in the left eye. Orbital computed tomography (CT) imaging revealed an enlargement of the left frontal sinus attributed to a 12 × 26 × 34 mm, well‐defined, hyperdense mass originating from the sinus floor (orbital roof). This mass displaced the left inferior globe, causing dystopia, without evidence of bone erosion. Additionally, the scan revealed a well‐defined, expansile, lytic lesion situated in the left frontal sinus, characterized by marked enhancement but lacking sclerotic margins or evidence of cortical breakthrough (Figure 1). The biopsy was performed prior to the surgery, yielding inconclusive results.

**FIGURE 2:**
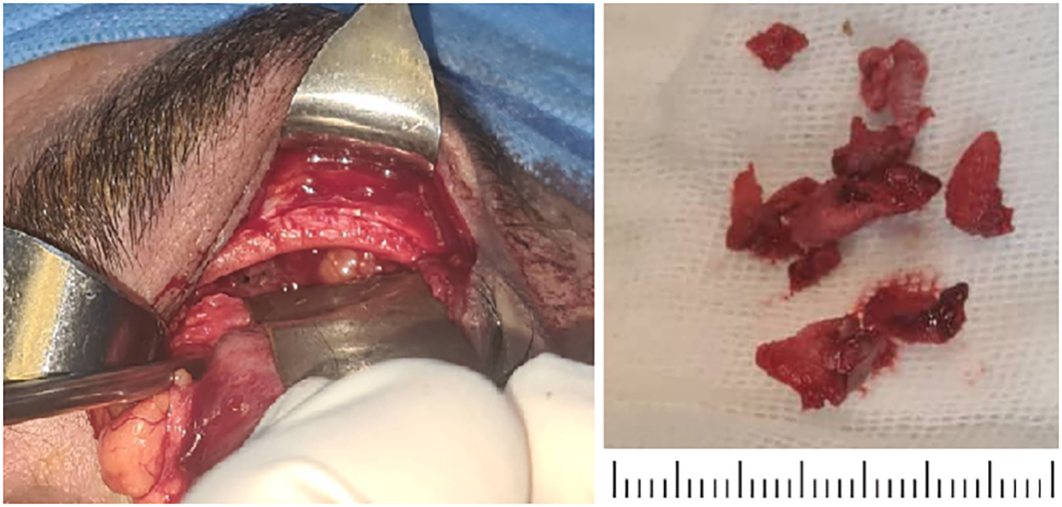
The image on the left depicts a mass during surgery in the orbital roof, and the image on the right shows the gross specimen, a creamy pink gritty mass that has been fragmented during the surgical procedure.

The diagnostic impression was a left frontal sinus fibro‐osseous tumor, leading to the patient's scheduling for surgery in a collaborative ENT and oculoplastic operation. Under general anesthesia, a superior eyelid crease incision was made, followed by suborbicularis dissection up to the superior orbital rim, exposing the bare bone of the anterior and inferior frontal sinus walls. An osteotomy through the sinus wall revealed a hemorrhagic, brittle, and fibro‐osseous mass. The mass was carefully excised in a piecemeal fashion by meticulous curettage, continuing until the dura mater was exposed (Figure 2). After complete removal of the mass, which necessitated total removal of the sinus floor and the opening of the normal sinus drainage medially, the surgical team ensured the absence of cerebrospinal fluid leakage from the dura. The surgery concluded with the suturing of the eyelid crease wound using Nylon 6‐0 sutures.

**FIGURE 3:**
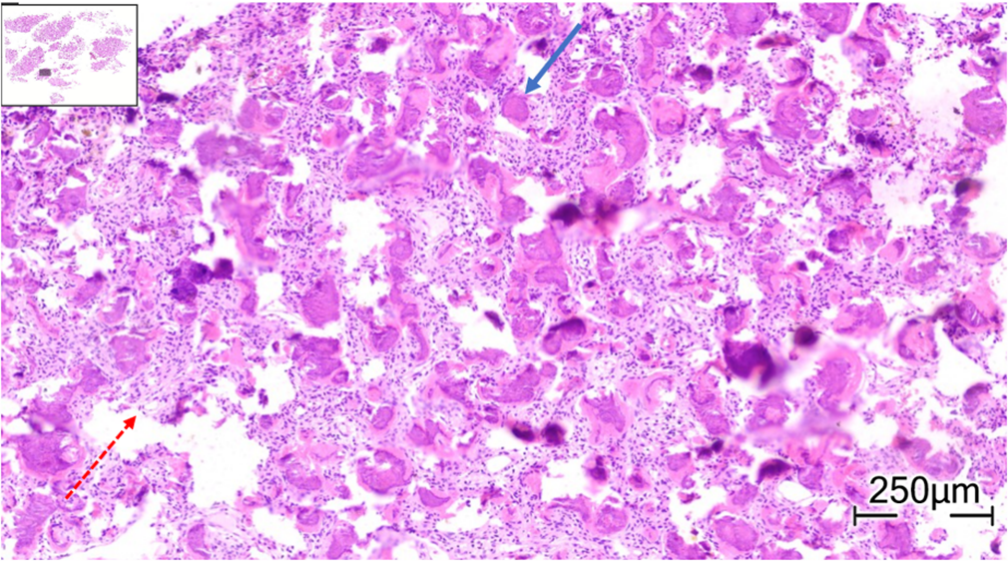
Hypercelluar low grade fibroblastic tumor, forming variable size of psommomatoid bodies. The blue‐solid arrow indicates psammoma‐like bodies, and the red‐dashed arrow points to spindle cell fibrous stroma.

The histopathological evaluation of the mass reported it as a hypercellular fibroblastic tumor with spindle cell morphology. Some of the cells exhibited hyperchromatic nuclei, with no mitosis observed, while others formed a mineral matrix with numerous calcified structures, referred to as psammomatoid ossifying fibroma (Figure 3).

Post‐surgery, the patient displayed no signs of restricted eye movement or pain, and his quality of life was assessed as good in terms of daily activities. No further treatment beyond surgical intervention was deemed necessary for the patient.

To provide a comprehensive understanding of the surgical procedure performed, we have included a (Video S1) within this manuscript, offering a visual representation of the operative techniques employed for reference and enhanced clarity.

## 3 DISCUSSION

Psammomatoid ossifying fibroma (POF) is a rare tumor affecting the craniofacial region, typically seen in teenagers and young adults, with most cases occurring between 16 and 33 years of age. However, it's worth noting that instances have been reported in individuals as young as 3 months and as old as 72 years, indicating a diverse age range of occurrence. Epidemiological studies indicate that POF tends to localize primarily in the maxillofacial area, with approximately 44% found in the maxilla and 56% in the mandible.^1^, ^2^ The uniqueness of our presented case of psammomatoid ossifying fibroma (JPOF) lies in both its exceptional location and the age of the patient. Unlike the majority of JPOF cases that predominantly manifest in the maxillofacial region of adolescents and young adults, our patient, at the age of 50, defies the conventional age range associated with this condition. Furthermore, the location of the JPOF within the left frontal sinus adds an additional layer of rarity, as such occurrences in paranasal sinuses are relatively infrequent. This atypical presentation underscores the importance of considering JPOF as a diagnostic possibility, even in older individuals and unusual anatomical sites, broadening the clinical spectrum of this enigmatic craniofacial lesion.

During the evaluation of the patient's clinical presentation, multiple differential diagnoses were considered due to the presence of left eye proptosis and a radiologically observed calcified mass in the left frontal sinus. These encompassed frontal sinus osteoma, mucocele, fibro‐osseous lesions, and frontal sinus malignancies, including recurrence of squamous cell carcinoma. However, these possibilities were systematically ruled out through a combination of clinical assessment, radiological evidence, and histopathological examination, with the diagnosis ultimately confirmed as psammomatoid ossifying fibroma (POF) of the frontal sinus.

The key radiological feature of ossifying fibroma is its evolution from primarily radiolucent in the early stages to a mixed density appearance as it enlarges, eventually encased in a thin radiolucent soft tissue capsule. It exclusively affects the jaws, often in the mandibular posterior region and anterior to the maxillary premolars. Unlike fibrous dysplasia, it has a distinct thin radiolucent border and concentric cortical expansion. It can displace nearby structures, notably the maxillary sinus, and appears with varying signal intensities on MRI, aiding in its differentiation from other lesions. These characteristic radiological traits are vital for accurate diagnosis and management.^3^ Cemento‐ossifying fibroma (COF) can cause tooth displacement or root resorption. In contrast, juvenile variants like juvenile trabecular ossifying fibroma (JTOF) and juvenile psammomatoid ossifying fibroma (JPOF) exhibit more aggressive growth patterns, potentially leading to dehiscence along expanded outer cortices, impinging on nearby structures. JPOF, frequently located in the sinuses, especially the ethmoid sinus, and presenting numerous small psammomatoid bodies on radiographs, stands out due to its distinct clinicopathological characteristics, making it essential to differentiate these variants during evaluation.^4^

Histopathological evaluation is crucial for psammomatoid ossifying fibroma (POF) to confirm the diagnosis, differentiate it from similar lesions, assess its cellular characteristics and aggressiveness, identify atypical features, and contribute to research and documentation, enhancing our understanding of POF. It complements radiological findings for a comprehensive evaluation. Macroscopically, ossifying fibromas can vary from well‐defined, encapsulated masses with a yellow‐white gritty cut surface in cemento‐ossifying fibroma (COF) to typically non‐encapsulated, fragmented masses in juvenile trabecular ossifying fibroma (JTOF) and juvenile psammomatoid ossifying fibroma (JPOF). Microscopically, COF presents with variably hypercellular fibrous tissue and mineralized areas with osteoblastic rimming, whereas JTOF lacks osteoblastic rimming and shows cellular osteoid trabeculae. JPOF is characterized by numerous small ossicles known as psammomatoid bodies, which can coalesce into larger mineralized regions, sometimes with cystic degeneration, distinguishing it from other ossifying fibromas.^4^

The genetic profile of ossifying fibroma and its variants, including psammomatoid ossifying fibroma (POF), remains incompletely understood. Mutations in the CDC73 gene have been found in some patients with hyperparathyroidism‐jaw tumor (HPT‐JT) syndrome, linked to conventional cemento‐ossifying fibroma (COF). However, CDC73 mutations appear less relevant in sporadic COF cases. Research suggests a possible role for the MEN1 gene in COF development. Still, no consistent genetic marker has been identified. Limited data on the genetic basis of juvenile trabecular ossifying fibroma (JTOF) and juvenile psammomatoid ossifying fibroma (JPOF) include rare cases with specific chromosomal rearrangements in JPOF.^5^ CDC73 collaborates with the human PAF1 complex, histone methyltransferase complex, and RNA polymerase II to facilitate transcription elongation and 3′ end processing. In various malignancies like gastric, colorectal, ovarian, and head and neck cancers, a decrease in CDC73 expression has been identified using immunohistochemistry. This reduction in CDC73 expression aligns with more aggressive cancer characteristics and less favorable prognoses.^6^ The history of cecal adenocarcinoma in our patient, in addition to their psammomatoid ossifying fibroma condition, it may serve as a potential starting point for further exploration of genetic relationships in future research.

In the treatment of ossifying fibromas in the paranasal, frontal, and orbital sinuses, endoscopic resection is the preferred method. However, it should be noted that due to the presence of vascular proliferation within the tumor, the volume of bleeding can be relatively significant. Therefore, meticulous planning for the management of bleeding and preparedness to address it should be in place.^7^ The use of radiotherapy is not advisable due to the risk of malignant changes and potential complications later in life.^8^

In a subset of patients, tumor relapse is observed. Recurrence rates, as reported in cross‐sectional investigations, exhibit considerable heterogeneity, spanning from 6.7% to 28.6%.^9^, ^10^ Concretely, the investigation revealed a substantial association between certain variables and the recurrence of ossifying fibroma (OF) within the pediatric nasal–skull base context. Specifically, a history of prior surgical procedures, instances of significant intraoperative hemorrhage, characterized by blood loss surpassing 1000 mL, and the presence of tumors encroaching upon the sphenoid and/or sella turcica and clivus regions exhibited a statistically significant correlation with the reappearance of ossifying fibroma (OF). These outcomes substantiate the assertion that individuals with a surgical history, those encountering notable intraoperative blood loss, and those afflicted with tumors infiltrating critical skull base anatomical structures face an elevated risk of ossifying fibroma (OF) recurrence subsequent to surgical intervention.^11^

In conclusion, this unique case report sheds light on the diverse clinical presentations and diagnostic challenges posed by psammomatoid ossifying fibroma, emphasizing the importance of accurate diagnosis and multidisciplinary collaboration in managing this rare craniofacial anomaly. Further research is warranted to explore the genetic underpinnings and optimal management strategies for this intriguing condition.

## AUTHOR CONTRIBUTIONS

**Pegah Babaheidarian:** Data curation (equal); investigation (equal); project administration (equal). **Parisa Mokhles:** Data curation (equal); investigation (equal). **Saleh Mohebbi:** Resources (equal). **Razieh Shahnazari:** Data curation (equal); investigation (equal); project administration (equal). **Nasser Karimi:** Data curation (equal); resources (equal). **Donya Ghazinia:** Resources (equal). **Sina Karaji:** writing ‐ original draft (equal); Validation (equal); writing ‐ review and editing (equal); visualization (equal). **Shahriar Shirzadi:** writing ‐ review and editing (equal).

## CONFLICT OF INTEREST STATEMENT

The authors have stated explicitly that there are no conflicts of interest in connection with this article.

## ETHICS STATEMENT

The patient provided written informed consent for the publication of this case report and accompanying images.

## Supporting information


**Video S1.** Surgical precision: frontal sinus tumor excised via transorbital approach, with a superior blepharoplasty Incision preceding precise orbit dissection. Gradual subperiosteal flap release from the orbital margin, safeguarding eye muscles, superior rectus, and superior oblique. Subsequent osteotomy ensures gentle tumor extraction.

## Data Availability

The data that support the findings of this study are available on request from the corresponding author. The data are not publicly available due to privacy or ethical restrictions.
